# The Effect of Social–Emotional Learning Programs on Elementary and Middle School Students’ Academic Achievement: A Meta-Analytic Review

**DOI:** 10.3390/bs15111527

**Published:** 2025-11-10

**Authors:** Yuyang Zhao, Biao Sang

**Affiliations:** 1 Department of Social Work, School of Sociology and Political Science, Shanghai University, Shanghai 200444, China; 2 Lab for Educational Big Data and Policymaking, Ministry of Education, Shanghai 200234, China

**Keywords:** meta-analysis, elementary school, middle school, academic performance, social and emotional learning (SEL)

## Abstract

This meta-analysis summarized the effects of universal and targeted social and emotional learning (SEL) programs in 22 studies with 24,510 elementary and middle schoolers between 2011 and 2021. It is critical to note that the evidence base was dominated by elementary school research (20 studies), with findings for middle school students derived from only two studies. The current study focused on three main issues: (a) the effectiveness of SEL programs on students’ overall academic performance as well as in specific subjects; (b) possible moderators that could differentiate the overall effectiveness of SEL programs; and (c) possible moderators that presented different effects in different grade levels and subject areas. The results of this review indicated that SEL interventions had a positive effect on overall academic achievement (g = 0.08), elementary school students (g = 0.075), middle school students (g = 0.122), English language arts (g = 0.07), mathematics (g = 0.08), science (g = 0.06), and GPA (g = 0.33) compared to alternative interventions or standard practice. Subgroup analysis was performed with several moderators (i.e., student SES, intervention design, grade level, subject area, and report type). A significant difference was found based on intervention design, with quasi-experimental studies showing larger effect sizes than randomized controlled trials (RCTs). The categorical moderation analysis was performed based on the student’s grade level and subject area and found significant differences. Overall, SEL programs with quasi-experimental designs might be more beneficial for promoting students’ academic performance. Given the limited evidence, conclusions regarding middle school students are preliminary, and more studies on middle school students and students’ science performance are needed.

## 1. Introduction

“21st-century skills” have received growing consideration from business leaders, politicians, and educators, with a strong emphasis on social and emotional competencies alongside traditional academic skills (Saavedra & Opfer, 2012; Trilling & Fadel, 2009). There is now a broad consensus that schools are responsible for fostering students’ holistic development, which integrally includes their social and emotional growth alongside cognitive development (Collaborative for Academic, Social, and Emotional Learning [CASEL], 2025; Greenberg et al., 2017). Social and emotional skills are referred to as the critical components of the 21st century and employability skills (Trilling & Fadel, 2009), and they are critical for individuals’ development (i.e., personal and professional) and function in society (National Academy of Sciences, 2012). National and international empirical evidence indicates that students’ social and emotional skills positively related to their mental health (Ciarrochi et al., 2003; Weare, 2010), interpersonal relationships (dos Santos & Galli, 2017; Manusov et al., 2020), positive adjustment (Domitrovich et al., 2017), and collaboration (Abry et al., 2017; Schonert-Reichl et al., 2017).

Therefore, empirical research on such interventions has been conducted in many countries, such as the US (Bowles et al., 2017; McCormick et al., 2021), the UK (Berry et al., 2016; Panayiotou et al., 2019), Australia (Bowles et al., 2017), Japan (Nakano et al., 2021), Brazil (McCoy et al., 2021), and China (Kam et al., 2011) have endeavored to implement intervention programs that aimed to enhance students’ social and emotional skills in general. Similarly, countries like Sweden, Italy, and Spain are increasingly integrating SEL into their educational policies and practices, reflecting a pan-European recognition of its importance for holistic student development (Cefai et al., 2018). Meanwhile, studies are conducted for specific student groups, such as English language learner (ELL) students (Castro-Olivo, 2014; Cho et al., 2019), gifted students (Neihart, 2021; Van Tassel-Baska et al., 2021), and students with disabilities (Cavioni et al., 2017).

Even though the overarching quantitative synthesis of the effectiveness of social and emotional learning (SEL) programs is presented (e.g., Corcoran et al., 2018; Durlak et al., 2011; Payton et al., 2008), the effectiveness of SEL programs in elementary and middle school students’ overall academic performance, as well as possible differences among different subject areas, remains unclear. Therefore, we presented a meta-analysis to investigate the effectiveness of SEL programs on elementary and middle school students’ academic performance. We also provide valuable insights into possible differential effects of programs by including several related student characteristics (i.e., students’ socioeconomic status), study characteristics (i.e., intervention design and report type), and academic characteristics (i.e., grade level and subject areas) in a moderator analysis. The outcomes of this study may benefit a wide range of stakeholders such as teachers, educational policymakers, and scholars, who are interested in utilizing SEL programs.

### 1.1. Social and Emotional Learning

Students’ social and emotional skills are defined in several ways. The most cited definition was from the CASEL. CASEL defines SEL as “the process through which all young people and adults acquire and apply the knowledge, skills, and attitudes to develop healthy identities, manage emotions and achieve personal and collective goals, feel and show empathy for others, establish and maintain supportive relationships, and make responsible and caring decisions” (Collaborative for Academic, Social, and Emotional Learning [CASEL], 2025, para. 1). CASEL further identified five core competencies involved in SEL: self-awareness, self-management, social awareness, relationship skills, and responsible decision-making (Weissberg et al., 2013). Meanwhile, in recent years, the Organization for Economic Cooperation and Development (OECD) has also advocated for students’ social and emotional skills and conducted an international survey that assessed 10- and 15-year-old students’ social–emotional skills in many countries. The OECD refers to social and emotional skills as “the abilities to regulate one’s thoughts, emotions, and behavior” (OECD, 2021, p. 4). The OECD’s Survey on Social and Emotional Skills (SSES) was based on the big five personality traits. It involved five general skill categories (i.e., task performance, emotional regulation, collaboration, open-mindedness, and engaging with others) that can be further split into 15 lower-order skills and another compound skill, which involved critical thinking, meta-cognition, and self-efficacy. Compared with the CASEL’s SEL, the OECD’s SSES focused on international assessment and formed international comparisons rather than developing evidence-based interventions for children and adolescents. Therefore, the current review focused on CASEL’s SEL and its related skills as well as programs.

### 1.2. Rationale for Focusing on Elementary and Middle School

The decision to include students from elementary and middle school in the present meta-analysis is grounded in developmental theory and the scope of SEL frameworks. This period, encompassing early childhood to early adolescence, represents a critical and continuous sequence in the development of social and emotional competencies. According to Erikson’s psychosocial stages, this span covers the “Industry vs. Inferiority” stage (roughly ages 5–12), where children develop a sense of competence through school achievements, and the initial phase of “Identity vs. Role Confusion” (ages 12–18), where adolescents begin to form a cohesive self-identity (Erikson, 1963). The CASEL framework posits that the core competencies of self-awareness, self-management, social awareness, relationship skills, and responsible decision-making are developed and refined progressively throughout this entire K-8 period (Weissberg et al., 2015).

While acknowledging developmental differences—such as the shift from more concrete, adult-guided emotional regulation in early childhood to more complex, peer-influenced relationship management in early adolescence (Denham, 2006)—the fundamental SEL skills targeted by interventions remain consistent in nature, even as their application becomes more sophisticated. The primary aim of school-based SEL programs is to systematically foster these core competencies across this developmental continuum. By including the K-8 range, this meta-analysis seeks to provide a comprehensive overview of the academic impact of SEL during these foundational school years.

### 1.3. Social and Emotional Learning Programs in Elementary and Middle School

In response to the recognized importance of SEL, a diverse array of evidence-based programs has been developed for elementary and middle school settings. These programs vary in their theoretical underpinnings, scope, and implementation strategies but share the common goal of systematically fostering the CASEL core competencies. Universal, curriculum-based programs are designed for all students in a classroom or school. Among the most widely researched is the Promoting Alternative Thinking Strategies (PATHS) Curriculum, which is grounded in the Affective–Behavioral–Cognitive–Dynamic model of development and provides lessons on emotional literacy, self-control, and problem-solving (Hennessey & Humphrey, 2019). Similarly, the RULER Approach developed by the Yale Center for Emotional Intelligence teaches skills for Recognizing, Understanding, Labeling, Expressing, and Regulating emotions (Brackett et al., 2012). The 4Rs Program (Reading, Writing, Respect, and Resolution) integrates literacy development with SEL, using children’s literature to teach conflict resolution and emotional understanding (Jones et al., 2011). Second Step is another comprehensive curriculum that targets empathy, emotion management, and social problem-solving (Low et al., 2015). Other programs employ a multi-component or ecological approach, intervening at multiple levels. INSIGHTS into Children’s Temperament engages teachers, parents, and children, using a temperament framework to help adults better understand and support children’s individual differences (McCormick et al., 2021). The MindUP Program incorporates mindfulness-based practices to enhance self-regulation and focus (Schonert-Reichl et al., 2015). More detailed descriptions of SEL interventions could be found in Frye et al. (2022), Jagers et al. (2015), and Rimm-Kaufman (2015).

Implementing effective interventions that focused on students’ social and emotional skills or competencies has been highly beneficial for the development across multiple domains, including their emotion, behavior, academic performance, and others (Frye et al., 2022; Yang et al., 2020; Zins et al., 2004). For example, such interventions or programs have been found to exert a positive influence on elementary school students’ emotional regulation (de Carvalho et al., 2017; Green et al., 2021), resilience (Green et al., 2021; Yamamoto et al., 2017), problem-solving abilities (Green et al., 2021), problem behaviors (Wong et al., 2014), and academic achievement (Kopelman-Rubin et al., 2021; McCormick et al., 2015). They have also demonstrated benefits for middle school-aged students’ math achievement (Lemberger et al., 2018), academic disengagement (Moore McBride et al., 2016), physical aggression (Espelage et al., 2013), self-esteem (Coelho & Sousa, 2018), resilience (LaBelle, 2019), and academic achievement (Babalis et al., 2013; Dougherty & Sharkey, 2017). Moreover, researchers have found that the SEL program has a positive influence on diverse and low-achieving elementary and middle school-aged students’ social awareness and relationship skills (Lewis et al., 2021; Van De Sande et al., 2022) and bullying prevention among students with disabilities (Espelage et al., 2015). Additionally, SEL programs such as CASEL have been found to benefit both elementary and middle school-aged students in both in-school and after-school settings, and students with and without behavioral and emotional problems across multiple domains, including s attitudes toward self and others, connectedness to schools, prosocial behaviors, academic achievement, conduct problems, and emotional distress (Payton et al., 2008). However, the effectiveness of SEL programs is not always promising, especially in terms of academic achievement in different subject areas. One recent study found that SEL interventions did not make significant group differences in elementary students’ English language arts and mathematic achievement (Xia et al., 2022).

### 1.4. Previous Meta-Analytic Evidence for Social and Emotional Learning Interventions

Previous meta-analytic reviews generally supported the effectiveness of social and emotional skill interventions (e.g., SEL) on K-12 children’s and adolescents’ overall development. For example, one review in the field, conducted by Payton et al. (2008), which reviewed 324,303 kindergarten through eighth-grade students, concluded that SEL programs had a significant positive effect on students’ development of social and emotional skills, positive attitudes toward self, others, and school, social behaviors, conduct problems, emotional distress, and overall academic performance. Similarly, one of the most influential reviews related to SEL interventions was conducted by Durlak et al. (2011), who used 270,034 kindergarten through high school students to examine the effectiveness of school-based universal interventions. In general, Dulark and his colleagues found a significant positive effect on SEL skills (g = 0.57), attitudes (g = 0.23), positive social behavior (g = 0.24), conduct problems (g = 0.22), emotional distress (g = 0.24), and overall academic performance (g = 0.27). Regarding academic performance, Durlak and his colleagues further examined the different mean effects according to intervention format (i.e., teacher, non-school, personnel, multicomponent) and different moderators (i.e., recommended training procedures, reported implementation problems). Additionally, Corcoran et al.’s (2018) meta-analysis, focused on school-based SEL programs, found that SEL had a significant effect on prek-12 students’ reading (g = 0.25), mathematics (g = 0.26), and science (g = 0.19) by analyzing studies ranging from 1970 to 2016.

However, by reviewing these three influential and most-related reviews as well as other review articles (e.g., Clarke et al., 2015; Maynard et al., 2017; Sklad et al., 2012; Taylor et al., 2017), we proposed the following major concerns: first of all, the educational landscape for K-12 students has undergone significant transformations in recent years, necessitating an updated understanding of SEL’s efficacy. The proliferation of digital technology and the subsequent shifts in learning environments, accelerated by the COVID-19 pandemic, have profoundly impacted students’ social interactions, emotional well-being, and academic engagement (Grewal et al., 2022; Hamilton et al., 2020). Such emerging technology not only disrupts children’s cognitive processes like attention but also the distractions on the experience and regulation of emotions (Greening et al., 2021). These changes underscore the need for skills like self-regulation and relationship-building that SEL programs aim to cultivate. Furthermore, evolving pedagogical approaches in core subject areas, such as a greater emphasis on collaborative problem-solving in mathematics (Schmidt & Burroughs, 2020) and complex digital literacy in reading (Leu et al., 2020), align closely with SEL competencies. Therefore, an updated meta-analysis focusing on the past decade is critical to ascertain how SEL programs support academic achievement within these contemporary contexts. Secondly, most previous reviews focused on a wide range of grade levels (kindergarten through high school) rather than extensively focusing on elementary and middle school students, which limited our understanding of the effectiveness of SEL programs on this specific population. Elementary and middle school students demonstrated unique developmental characteristics in many aspects, such as cognitive, logical thinking, reasoning development (Larrain et al., 2021; Yunus, 2021), social, moral, and emotional development (Eisenberg, 2001; Erikson, 1963, 1968; Piaget, 1964; Rice & Dolgin, 2008). Lastly, most previous meta-analysis studies examined the influence of SEL programs on overall academic performance. However, different subject areas make unique cognitive and social–emotional demands on students (Fonteyne et al., 2017; Schmidt & Burroughs, 2020; Vedel et al., 2015). For instance, mathematics often requires perseverance and managing frustration when solving difficult problems (Galla et al., 2014), while science demands collaboration and critical thinking (Osborne, 2014). Therefore, investigating subject-specific effects is not merely analytical refinement but a necessity for understanding how SEL competencies translate into academic gains across distinct domains. This approach can provide educators with more precise guidance on tailoring SEL supports to enhance learning in specific subjects.

### 1.5. The Present Study

Building upon the existing literature, the purpose of the current review was to systematically analyze the effectiveness of SEL programs on students’ academic performance in elementary and middle schools separately, and it focused on three issues: (1) the effect of SEL programs on students’ overall academic performance as well as on performance in specific subject areas (i.e., English, language arts, mathematics, science, and GPA); (2) explored if these effects were moderated by student-related factors (i.e., students’ SES), study-related factors (i.e., intervention design and report type), and academic-related factors (i.e., grade level and subject area); (3) investigated the effectiveness of different moderators in their ability to alter the effects of SEL programs in different grade level and subject areas. By addressing these aims, our study updates the evidence base with recent studies, provides much-needed specificity regarding subject-area effects, and directly tests for differential impacts across the critical developmental period of elementary and middle schooling.

## 2. Materials and Methods

### 2.1. Article Selection

Following the PRISMA guidelines (Page et al., 2021), a computer-based search of electronic research databases for empirical studies between 2011 and 2021 for SEL programs was conducted. More specifically, the current study utilized the following strategies to locate the potential studies included in the current review. First, we searched several databases: Academic Search Complete, Academic Search Elite, Academic Search Premier, Education Full Text (H.W. Wilson), ERIC, APA PsycARTICLES, APA PsycINFO, Web of Science, and Google Scholar. The following search terms and their variants were used: social–emotional skills, social–emotional learning, SEL, social–emotional competence, social–emotional intelligence, social–emotional programs, psychosocial, social skills, emotional intelligence, academic achievement, elementary school, middle school, primary school, secondary school, and interventions. These search terms were crossed with the age group of interest (children* and adolescents*) and design of study (intervention, quasi-experimental study). Second, we searched the possible SEL programs by using program names (e.g., PATH, INSIGHTS, RULLER, 4Rs, and MindUP). Third, we searched for potential gray literature produced by organizations that promote SEL (e.g., CASEL, OECD, Yale Center for Emotional Intelligence, and the Partnership for 21st Century Learning). Fourth, some SEL program developers or researchers were contacted through email to ask for additional missing studies or data. Last, we utilized the snowballing method to find additional relevant studies that were not in the databases but referenced in the selected articles. The literature search was completed between 4 January 2022, and 25 February 2022.

The scope of the review was limited to elementary and middle school students (grades K-8). This range was selected because it represents a coherent educational block in many educational systems where universal SEL interventions are most frequently implemented, targeting the continuous development of core social–emotional competencies (Weissberg et al., 2015). To address the substantial developmental span within this range, we pre-specified a moderator analysis by grade level (elementary vs. middle school) to examine potential differential effects.

### 2.2. Inclusion Criteria

Studies with the following characteristics were selected:

1. Any program around the world with a language available in English.
2. Appeared in publication (including peer-reviewed papers or non-peer-reviewed reports) from 1 January 2011 to 31 December 2021.
3. Exclusive to elementary or middle school students receiving a universal or targeted SEL intervention using SEL conceptual frameworks or relevant theories.
4. Included a control or comparison group that could compare students who received an intervention that targeted SEL skills as categorized by CASEL (self-management, self-awareness, social awareness, responsible decision-making, and relationship skills) with students who received an alternative intervention or standard practice.
5. Employed an RCT design or rigorous quasi-experimental design.
6. Reported sufficient statistical information to calculate the proper effect size at the post-test.
7. The outcome measures included quantitative indicators of students’ academic performance: such as English language arts, mathematics, and science.

### 2.3. Exclusion Criteria

Studies with the following characteristics were excluded from the study:

1. Studies did not specifically report outcomes during the elementary or middle school years.
2. Studies focused primarily on outcomes related to physical well-being, such as health, nutrition, exercise programs, and programs to prevent drug use and pregnancy.
3. Studies employed single-group, single-case, or non-equivalent quasi-experimental designs; studies did not examine preexisting group differences.
4. Studies had small-group out-of-class programs (i.e., each condition had less than 15 students) to avoid confounding treatment effects with potential teacher effects.

### 2.4. Screening Procedure

**Figure 1:**
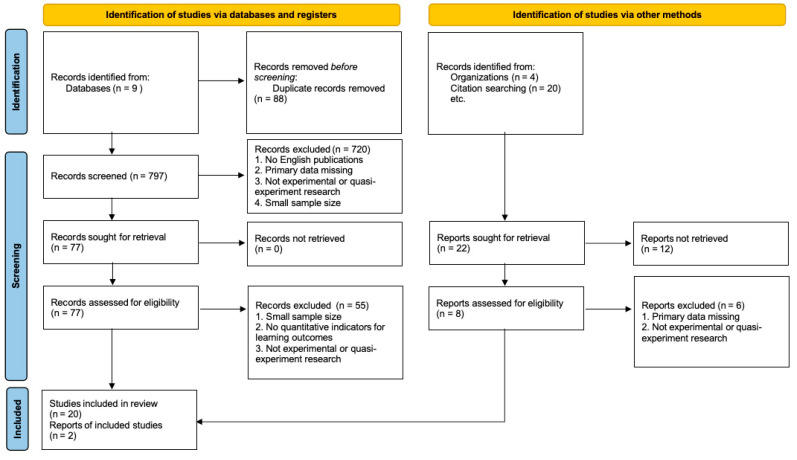
PRISM 2020 diagram.

The current study followed the review protocol and guidelines for screening relevant studies based on the Preferred Reporting Items for Systematic Reviews and Meta-Analyses (PRISMA) 2020 (Page et al., 2021). As shown in Figure 1, 877 studies were initially identified from the selected databases, program reports, and snowballed method, which were exported to Endnote X9 for further screening. After removing the 80 duplicate studies, 797 studies remained. Out of them, seven hundred twenty studies were further removed based on the title and abstract reading. The final stage of screening included 77 full-text assessable studies. The more detailed reading led to the exclusion of 55 studies since they had a small sample size, did not report any quantitative indicators for the outcome variables, or did not use RCT or non-equivalent quasi-experimental designs. Thus, the final 22 studies were included in the meta-analysis, consisting of 17 universal studies and five targeted studies. All screening processes were completed by the first two authors of the study.

### 2.5. Coding Procedure

A coding procedure was followed based on the recommendations by Cooper (2015): (1) identifying the information for the study and coders; (2) summarizing the description of the studies’ SEL interventions; (3) organizing the description of the studies’ sample/participants; and (4) coding the studies’ outcomes (i.e., effect size information). To understand potential factors that might contribute to the effectiveness of SEL programs, we extracted information about the studies’ design and potential moderators (i.e., students’ SES, intervention design, grade level, subject area, and report type). More specifically, students’ SES was coded as low SES (greater than 50% of the students qualified for free or reduced-price meals), medium or high SES (equal to or less than 50% of the students qualified for free or reduced-price meals), and unknown (studies did not indicate students’ SES). The intervention design was coded as either an RCT (random control trial) or quasi-experimental design. The grade level was coded as either elementary school or middle school. The subject area was coded as English language arts, mathematics, science, or GPA. More specifically, English language arts involve academic achievement in reading, writing, English, and other languages achievements. The report type was coded as either peer-reviewed articles or reports. We extracted the information needed to calculate the effect size in the last step. We contacted the researchers for additional missing data to calculate the effect size.

The first two authors of the study were the coders, who first coded the same three (around 10% of the total studies) articles independently to see if there were any discrepancies between the two coders. Discrepancies and issues noticed in the coding process were discussed and resolved through the discussion with the research team. No major discrepancies were observed in the final coding process. The interrater agreement was 0.91.

### 2.6. Effect Size Calculations and Statistical Analysis Method

We follow the recommendations and techniques proposed by Cooper et al. (2019) and Lipsey and Wilson (2001) to calculate effect sizes. Generally, from each primary study, standardized mean differences (Cohen’s d) were either extracted or computed for each outcome of interest. The nested data structure was considered when extracting effect sizes from primary studies since they were intervention studies. When studies employed hierarchical linear models, effect sizes were extracted from the model to account for cluster randomization. After all effect sizes were extracted in the form of Cohen’s d, Hedges’s g was computed and used as the effect size for all analyses.

From the 22 studies included in the meta-analysis, a total of 78 effect sizes were extracted. Thus, there were multiple outcomes and multiple types of measurement (e.g., student achievement tests and teacher-reported achievement) involved in one study. To deal with these multiple effect sizes, multiple outcomes were entered for each study and then averaged. All analyses were performed using the Hedges and Olkin approach to meta-analysis. The random-effects model was selected since we assumed that the population effect size would not be the same for every study. The analysis was conducted using the Comprehensive Meta-Analysis software (V3) (CMA, Borenstein et al., 2013). CMA calculates the effect sizes, study variances, and heterogeneity among studies. We employed the trim-and-fill analysis to evaluate publication bias (Duval & Tweedie, 2000). Finally, we used subgroup analysis with potential moderators to account for the observed heterogeneity.

## 3. Results

### 3.1. Descriptive Characteristics of Reviewed Studies

Of the 827 initially screened studies, 22 qualified studies contained 76 effect sizes, and 24,510 elementary and middle schoolers were included in the meta-analysis. Of the 22 studies, 20 studies were on elementary schools (comprising the vast majority of the evidence base) and only two studies were on middle schools; seventeen studies were on universal interventions and five studies were on targeted interventions; 17 studies included dependent measures of the language of arts performance (i.e., reading, writing, English, Hebrew) (*N* = 24,638); 15 studies included dependent measures of mathematic performance (*N* = 24,590); 5 studies included dependent measures of GPA/Aggregate Score (*N* = 3416); only one study included dependent measures of science performance (*N* = 6118). The primary studies consisted of 20 peer-reviewed studies and two reports. Table 1 provides descriptive information for each study included.

### 3.2. Overall Effect

As shown in Table 2, compared with students in control conditions, students who received SEL interventions showed higher overall academic achievement (*n* = 22, g = 0.08, 95% confidence interval [CI] = [0.05, 0.12]). The Q-value of 238.51 was also significant (*p* < 0.01), and I2, indicating the true variance, was at a moderate to a high level (68.55), suggesting that most of the variance in effect sizes represents true variance. The mean effect sizes for mathematics, English language arts, science, and GPA were 0.08, 0.07, 0.06, and 0.33, respectively. Because the science and GPA outcome data came from only three and six sources, some caution should be exercised when interpreting such results. Except for science, the I2 for mathematics, English language arts, and GPA were at a moderate to high level (65.25–86.73).

Given the developmental and contextual differences between elementary and middle schools, we also computed overall effect sizes separately for each group. The effect size for elementary school students (k = 66, g = 0.075, SE = 0.019) was similar to that for middle school students (k = 10, g = 0.122, SE = 0.024). However, the middle school result is based on only 2 primary studies (yielding 10 effect sizes) and thus has high uncertainty that should be interpreted with caution; the difference between grade levels was not statistically significant (Q = 2.338, *p* = 0.126).

### 3.3. Sensitivity Analysis

The current study conducted a sensitivity analysis to see if extreme outliers might influence the results. By conducting the one-study removal analysis (Borenstein et al., 2009), the results showed that there was a range of effect sizes that fell within the 95% confidence interval of the overall effect size (0.04 to 0.12, 0.02 to 0.13, −0.38 to 0.72, and 0.05 to 0.76 for overall effect size, mathematics, English language arts, and GPA, respectively). Therefore, these results indicated that it would not impact the overall effect size if one effect size were removed except for English language arts.

### 3.4. Publication Bias

**Figure 2:**
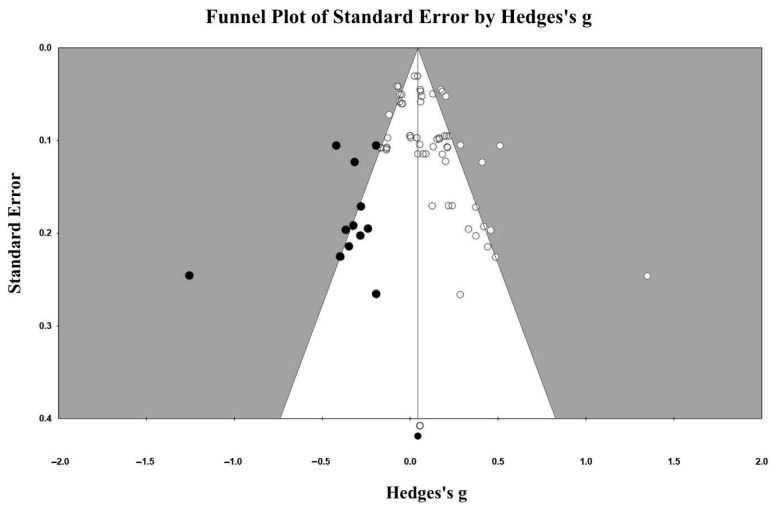
Funnel polit with imputed studies.

We employed the trim-and-fill analysis (Duval & Tweedie, 2000), a procedure to correct the combined effect size estimate, to examine the potential publication bias among studies included in the meta-analysis. This analysis identifies the missing studies and their effect sizes. As shown in Figure 2, our analysis revealed that no missing studies were identified and needed to be added, resulting in the same adjusted point estimate and confidence interval as the main results (d = 0.046, 95% CI = [0.011, 0.084]). The confidence interval for the true effect did not include zero after imputation. Thus, this analysis showed no evidence of publication bias.

### 3.5. Categorical Moderation Analysis

Using subgroup categorical moderator analysis (Borenstein et al., 2009), we calculated effect sizes and 95% CI for each level of five categorical moderators. The overall results and all between-group heterogeneity tests are displayed in Table 3. Of all five variables tested, only one moderator, intervention design, was significant (*p* = 0.013). More specifically, the mean effect size for the quasi-experimental study (k = 12, g = 0.27, SE = 0.082) was significantly larger than that for randomized studies (k = 64, g = 0.063, SE = 0.015). However, this finding must be treated with caution given the many study differences between these two groups.

Moreover, studies involving low SES (k = 40, g = 0.056, SE = 0.021) did not significantly differ from studies involving medium/high SES (k = 13, g = 0.085, SE = 0.048) and unknown SES (k = 23, g = 0.126, SE = 0.026). There was also no significant difference among different subject areas, such as English language arts (k = 34, g = 0.074, SE = 0.026), mathematics (k = 33, g = 0.072, SE = 0.024), science (k = 3, g = 0.061, SE = 0.028), and GPA (k = 6, g = 0.326, SE = 0.105). Finally, no significant difference was found between different report types, peer-reviewed articles (k = 65, g = 0.082, SE = 0.02), and reports (k = 11, g = 0.082, SE = 0.031).

Given the substantial disparity in the evidence base, we report overall effects separately by grade level. For elementary school students (k = 20), the overall effect was significant and positive (g = 0.075, 95% CI = [0.038, 0.112]). For middle school students (k = 2), the overall effect was also positive (g = 0.122, 95% CI = [0.075, 0.168]) but must be interpreted with extreme caution due to the very small number of contributing studies, which limits the precision and generalizability of this estimate.

### 3.6. Categorical Moderation Analysis Based on Student’s Grade Level

We further examined SEL intervention effects based on students’ grade levels (elementary and middle school). The current study involved 66 effect sizes for elementary school and 10 for middle school.

Regarding elementary school, as presented in Table 4, two of the four moderators reached significant levels: intervention design (*p* = 0.14) and report type (*p* = 0.14). More specifically, the mean effect size for the quasi-experimental study (k = 11, g = 0.26, SE = 0.085) was significantly larger than that for RCT studies (k = 55, g = 0.043, SE = 0.017). The mean effect size for peer-reviewed articles (k = 64, g = 0.082, SE = 0.02) was significantly larger than that for reports (k = 2, g = −0.07, SE = 0.029). However, this finding must be treated with caution given the large number of study differences between these two groups. Regarding middle school, as shown in Table 5, none of the four moderators reached a significant level.

### 3.7. Categorical Moderation Analysis Based on the Subject Area

Last, we examined students’ performance in different subject areas (i.e., mathematics and English language arts) to understand the variation in the models. Even though four subject areas were involved in the main analysis, we only chose mathematics and English language arts performance because there were only three and six effect sizes for students’ science and GPA performance, respectively.

Regarding students’ mathematical performance (Table 6), only students’ SES showed group differences (Q = 8.221, *p* = 0.016). Regarding students’ English language arts performance (Table 7), the mean effect size for quasi-experimental study (k = 12, g = 0.27, SE = 0.082) was significantly (*p* = 0.013) larger than that for RCT studies (k = 65, g = 0.063, SE = 0.015).

## 4. Discussion

Built upon previous studies on the effectiveness of SEL programs, the current meta-analysis increased our understanding of SEL programs’ effect on students’ academic performance in several ways, both in terms of updating and extending the findings from previous reviews on the effect of SEL programs, and also in terms of shedding light on the effects of several moderators on this effectiveness that were not known previously. All relevant findings and new insights were discussed in the following sections.

### 4.1. The Overall Effect of SEL Programs

The current meta-analysis reviews the empirical studies and reports on the effects of SEL programs on students’ overall and specific subject academic achievement in elementary and middle school, and findings partially consistent with previous meta-analyses indicate SEL programs have a significant and positive influence on student overall academic achievement (e.g., Corcoran et al., 2018; Durlak et al., 2011; Payton et al., 2008). In addition, by reviewing the recent ten years of SEL programs, the current review provided new insights into how SEL programs affect students’ academic achievement in different subjects. More specifically, the current review found SEL programs not only have a significant and positive effect on mathematics and science (e.g., Corcoran et al., 2018), but also on English language arts and GPA. Even though the effect size in the present review (e.g., ES = 0.08) was smaller than those in the previous reviews (e.g., ES = 0.27), as recently suggested by Kraft (2020), in the field of education, the effect size benchmark for evaluating effects on student achievement among elementary, middle, and high school student is “less than 0.05 is small, 0.05 to less than 0.2 is medium, and 0.2 or greater is large” (p. 18). Based on these rules of thumb, the findings from the current study suggest a significantly positive and medium effect on elementary and middle school students’ overall academic performance, mathematics, English language arts, and science, and a significantly positive and large effect on their GPA. This effect, while statistically significant, is considered small. However, even small effects can be meaningful when applied at scale across an entire school system. For instance, an effect size of 0.08 could translate to moving an average student from the 50th to the 54th percentile, representing a meaningful boost in learning over time. Our study confirmed the general finding that well-designed school-based SEL programs can be an effective way to improve students’ academic performance.

### 4.2. Moderating Effects of Student Characteristics

As indicated by previous researchers that student diverse characteristics and their intersections may be related to program outcomes (Garner et al., 2014), the current review further examined the moderating effects of student characteristics, namely student SES. Similarly to Corcoran et al.’s (2018) findings, the results from the current review revealed that student SES was not a significant moderator in general, which indicated there was no evidence that one group benefited more than another. However, when testing its moderating effect according to grade level and subject area, we found that student SES was a significant moderator in students’ mathematical achievement. Even though it reached a significant level, such a group difference was mainly due to the difference between the unknown group and the other two groups (i.e., low SES and medium or high SES). Therefore, such results must be interpreted with caution. Since the previous study found that the moderating effect of student SES on SEL programs is neither promising nor lacking (Rowe & Trickett, 2018), more detailed reviews are needed to test its effect.

### 4.3. Moderating Effects of Study Characteristics

Similarly to Corcoran et al.’s (2018) findings, our results further revealed that true experiments produced significantly smaller effect sizes than quasi-experiments in general, which provided empirical evidence for the idea that RCT research in education often produces lower effect sizes than that in quasi-experiments (Cheung & Slavin, 2016). Our review further proved that such significant differences were also presented in elementary school and in English language arts, but not in middle school or in mathematics. However, such findings must be treated with caution given the relatively small sample size for quasi-experimental designs.

On the other hand, even though the report type, namely peer-reviewed articles or reports, was not found to be a significant moderator in general. Even though no significant group differences were found between the peer-reviewed article and the unpublished report, the effect size for the peer-reviewed article was much larger than that of the unpublished report, which aligns with the previous study (Cheung & Slavin, 2016). Regarding different grade levels and subject areas, a significant group difference was found only in the elementary sample. However, the extremely small sample size for the report must be noticed.

### 4.4. Moderating Effects of Academic Characteristics

Finally, the current study examined the effect of potential moderators in different grade levels and subject areas. The quasi-experimental design in elementary schools showed a significantly larger effect than the RCT design, and peer-reviewed articles showed a significantly larger effect than reports. Whereas in middle schools, none of the moderators showed a significant difference. This lack of significant findings is likely due to the very small number of studies available for middle school students (k = 2), which severely limits the statistical power to detect moderator effects. Therefore, the results from the present study indicated that research design matters when considering the effect of SEL programs on elementary students’ academic performance. One possible explanation could be the differences between quasi-experimental and RCT designs. The heightened internal validity of RCTs often comes with practical constraints that may dampen effect estimates. In the context of SEL, the very nature of the intervention—fostering relational trust, safety, and prosocial skills—may be particularly sensitive to these research conditions. The structured protocols and monitoring in RCTs can sometimes reduce the flexibility for facilitators to be fully responsive to group dynamics, potentially diluting the program’s impact (Durlak et al., 2011). Furthermore, the act of randomization itself, especially at the individual level, can create groups that lack the pre-existing social cohesion found in intact classrooms, which are the typical unit of assignment in quasi-experimental designs. Building the trusting relationships essential for SEL may take longer or be more challenging in such newly formed groups, whereas interventions delivered to intact classrooms can build upon existing peer networks (Denham & Brown, 2010). This may be especially critical for younger, elementary school students whose social–emotional development is closely tied to stable and familiar environments.

Regarding students’ mathematical performance, even though SEL programs showed significant differences in the effect sizes of students’ SES, it was not mainly due to the difference between low and medium or high SES students. However, such a significant difference was not found in English language arts. A significant difference was found in intervention design regarding students’ English language arts performance, with quasi-experimental studies showing a higher effect size than RCT studies.

### 4.5. Implications and Limitations

There are several limitations to the current study. First, the current study employed quantitative measures for students’ academic performance; future researchers might want to employ more qualitative research because “qualitative studies can better understand the ‘black-box’ of SEL program interventions” (Corcoran et al., 2018, p. 69). Second, there were few studies in several subgroup analyses, such as studies conducted in middle school, quasi-experimental studies, and studies involving students’ science performance. The efficacy of SEL programs developed for children may not directly translate to adolescents, who require approaches that address their growing need for autonomy, identity exploration, and more complex peer relationships (Yeager, 2017). Extreme imbalance between elementary and middle school studies is a critical limitation. Our search yielded only two studies focusing exclusively on this developmental period, compared to twenty at the elementary level, precluding any firm conclusions about the effectiveness of SEL programs specifically for the middle school population. Third, it is important to note that all studies included in this meta-analysis were published between 2011 and 2021, prior to the widespread impact of the COVID-19 pandemic. The pandemic has profoundly altered educational contexts, with shifts to remote learning, reduced peer interaction, and heightened stress and anxiety among students (Erstad et al., 2024; Hamilton et al., 2020). These changes may influence both the implementation and effectiveness of SEL programs. For instance, some studies suggest that SEL interventions delivered in hybrid or remote formats may face challenges in fostering peer relationships and teacher–student connections (Yeager, 2017; Erstad et al., 2024). Conversely, the heightened focus on mental health during the pandemic may have increased the perceived relevance of SEL (Greenberg et al., 2017). Future research should examine whether the effects of SEL programs observed in pre-pandemic contexts hold in the post-pandemic era, particularly as schools continue to integrate digital and blended learning models. Fourth, while the primary focus of this meta-analysis was on academic achievement, it is worth noting that the benefits of SEL programs are theorized to extend beyond the classroom. The CASEL model posits that SEL competencies can foster positive outcomes across multiple settings, including the home and community (Weissberg et al., 2015). Empirical evidence suggests that SEL interventions can lead to improvements in students’ prosocial behavior, mental health, and relationship quality (Durlak et al., 2011), which may in turn positively influence their personal and family environments. Future research would benefit from directly assessing these ecological spillover effects to provide a more comprehensive understanding of SEL’s societal impact. Fiftieth, the current evidence base synthesized in this review is its predominant focus on programs implemented and evaluated in the United States, Australia, and the UK. Future meta-analytic work should make a concerted effort to systematically include and synthesize the growing body of non-English SEL research to provide a truly global perspective. Finally, the potential moderating role of participant gender was not examined in the present study due to insufficient reporting in the primary literature. We recommend that future primary studies consistently report and analyze outcomes by gender.

## 5. Conclusions

Previous studies have already demonstrated the effectiveness of SEL programs on students’ social, behavior, and academic performance (e.g., Durlak et al., 2011; Payton et al., 2008; Sklad et al., 2012). The present study builds on previous studies but extensively focuses on elementary and middle school students. We found positive and medium effects of SEL programs not only on their overall academic performance but also on individual subjects. The results provided empirical evidence and highlight the importance of implementing SEL programs in elementary and middle schools, especially for interventions targeting improving students’ academic performance. In addition, the current study emphasized the need to consider using quasi-experimental designs while implementing SEL programs that aim to increase students’ academic performance, where students can build SEL skills with acquainted peers and learn in a familiar environment, especially for elementary students and for improving students’ English language arts performance. The findings from the current study provided more specific and detailed results on the effect of SEL programs on elementary and middle school students’ mathematics and English language arts performance, which could help policymakers and practitioners select the best programs that will most benefit the students. The severe shortage of middle school studies makes it imperative for future research and funding to focus on this developmental stage to determine how SEL programs can be optimally designed and implemented for young adolescents.

## Figures and Tables

**Table 1:** Descriptive information for all included studies.

Study Name^1^	Country	Report Type	*N*	Design	Intervention Design	Tier	Grade	SES	Post-Test	Achievement Domain
Hennessey and Humphrey (2019)	UK	Peer-reviewed article	3336	RCT	PATHS	Universal	5, 6	Non-Low SES	Independent standardized testing (for year 5 post-test); Standardized Assessment Test (SAT) (for year 6 post-test)	English; Mathematics
McCormick et al. (2021)	USA	Peer-reviewed article	353	RCT	INSIGHTS	Universal	3, 4, 5, 6	Low SES	State English Language Arts (ELA); Math standardized test scores through administrative records	English (reading); Mathematics
Panayiotou et al. (2019)	UK	Peer-reviewed article	1626	Quasi-experimental	PATHS	Universal	3	Non-Low SES	2014 Key Stage 2 (KS2) national curriculum test scores	GPA
Hart et al. (2020)	USA	Peer-reviewed article	876	RCT	Social Skills Improvement System Class wide Intervention Program (SSIS-CIP)	Universal	3, 4, 5	Unknown	Pennsylvania System of School Assessment	Reading; Mathematics
Kopelman-Rubin et al. (2021)	Israel	Peer-reviewed article	419	Quasi-experimental	I Can Succeed-Elementary School (ICS-ES)	Universal	4	Non-Low SES	School Record	Hebrew; English; Mathematics
McCormick et al. (2015)	USA	Peer-reviewed article	433	RCT	INSIGHTS	Universal	1	Low SES	Teacher-reported	Reading; Mathematics
Schonfeld et al. (2015)	USA	Peer-reviewed article	705	RCT	PATHS	Universal	3, 4, 5, 6	Non-Low SES	State Mastery Test (MT).	Reading; Writing; Mathematics
Carroll et al. (2020)	Australia	Peer-reviewed article	854	Quasi-experimental	KooLKIDS	Universal	4, 5, 6	Non-Low SES	Teacher-reported	English; Mathematics
Tijms et al. (2018)	The Netherlands	Peer-reviewed article	90	RCT	bibliotherapeutic book club intervention	Targeted	6	Low SES	Vlaamse Test Begrijpend Lezen, version grade 6	Reading
Dougherty and Sharkey (2017)	USA	Peer-reviewed article	110	Quasi-experimental	Reconnecting Youth (RY)	Targeted	7, 9, 11	Low SES	School provided GPA	GPA
Brackett et al. (2012)	USA	Peer-reviewed article	273	Quasi-experimental	RULER	Targeted	5, 6	Non-Low SES	School report cards	English; Mathematics
Ashdown and Bernard (2012)	Australia	Peer-reviewed article	57	Quasi-experimental	You Can Do It! (YCDI)	Targeted	1	Low SES	Teacher-reported	Reading
Zhai et al. (2015)	USA	Peer-reviewed article	414	RCT	Chicago School Readiness Project Intervention	Universal	3	Low SES	Teacher-reported Academic Rating Scale (ARS).	Overall academic Skills; Language and literacy; Mathematical Thinking
Cook et al. (2018)	USA	Peer-reviewed article	4284	RCT	Second Step	Universal	1, 2	Low SES	Curriculum-based measurement (CBM) from Aimsweb website	Reading; Mathematics
O’Connor et al. (2014) S1	USA	Peer-reviewed article	106	RCT	INSIGHTS	Universal	1	Low SES	Letter–Word ID and Applied Problems subtests of the WJ-III	Reading; Mathematics
O’Connor et al. (2014) S2	USA	Peer-reviewed article	345	RCT	INSIGHTS	Universal	1	Low SES	The Academic Competency Evaluation Scale (ACES)	Language Arts; Mathematics
Jones et al. (2011)	USA	Peer-reviewed article	1184	RCT	4Rs Program	Universal	3	Low SES	Teacher reports; New York State standardized assessments of math and reading achievement	Academic skills (teacher-reported); Reading; Mathematics
Hanson et al. (2011)	USA	Report	2309	RCT	Tribes Learning Communities (TLC)	Universal	1, 2, 3, 4	Low SES	California administers the California Standards Tests (CST)	English language Art; Mathematics
Challen et al. (2011)	UK	Report	6118	RCT	Penn Resiliency Program (PRP)	Targeted	7	Unknown	National Pupil Database School records	English; Mathematics; Science
Schonert-Reichl et al. (2015)	Canada	Peer-reviewed article	99	RCT	MindUP program	Universal	4, 5	Non-Low SES	School records	Mathematics
Matsuba et al. (2021)	Northern Ugandan	Peer-reviewed article	82	Quasi-experimental	MindUP program	Universal	5, 6	Unknown	School records	Aggregate Score (English, Mathematics, social studies, and science)
McCormick et al. (2016)	USA	Peer-reviewed article	435	RCT	NSIGHTS	Universal	1	Low SES	Woodcock Johnson III Tests of Achievement, Form B (WJ-III)	Reading; Mathematics

**Table 2:** Effect sizes for overall and specific academic performance.

Random Model	k	ES	SE	Lower Limit	Upper Limit	Z-Value	*p*-Value	Q-Value	df (Q)	*p*-Value	I2
Main effect	76	0.08	0.02	0.05	0.12	5.03	00.000	234.77	73	0.000	68.91
Mathematics	33	0.08	0.02	0.03	0.12	2.98	00.003	92.48	32	0.000	65.40
English language arts	34	0.07	0.03	0.02	0.12	2.89	0.004	107.49	33	0.000	69.30
Science	3	0.06	0.03	0.01	0.12	2.22	0.027	0.02	2	0.099	0.00
GPA	6	0.33	0.11	0.12	0.53	3.12	0.002	30.48	5	0.000	83.60

Note: main effect involved all subject areas: mathematics, English language arts, science, and GPA.

**Table 3:** Subgroup categorical moderator analysis.

Moderator/Group	k	Point Estimate	Standard Error	95% CI [LL, UL]	Q-Value	df (Q)	*p*-Value
**Student SES**					4.238	2	0.12
Low SES	40	0.056	0.021	[0.015, 0.098]			
Medium or High SES	13	0.085	0.048	[−0.009, 0.179]			
Unknown	23	0.126	0.026	[0.074, 0.177]			
**Intervention Design**					6.22	1	0.013
Quasi-experimental	12	0.27	0.082	[0.11, 0.43]			
RCT	64	0.063	0.015	[0.033, 0.093]			
**Grade Level**					2.338	1	0.126
Elementary	66	0.075	0.019	[0.038, 0.112]			
Middle	10	0.122	0.024	[0.075 0.168]			
**Subject Area**					6.042	3	0.11
English language arts	34	0.074	0.026	[0.024, 0.124]			
Mathematics	33	0.072	0.024	[0.025, 0.119]			
Science	3	0.061	0.028	[0.007, 0.115]			
GPA	6	0.326	0.105	[0.121, 0.531]			
**Report Type**					0.008	1	0.93
Peer-reviewed	65	0.085	0.02	[0.047, 0.124]			
Report	11	0.082	0.031	[0.001, 0.022]			

**Table 4:** Subgroup categorical moderator analysis in elemental school.

Moderator/Group	k	Point Estimate	Standard Error	95% CI [LL, UL]	Q-Value	df (Q)	*p*-Value
**Students’ SES**					3.576	2	0.167
Low SES	39	0.052	0.021	[0.011, 0.093]			
Medium or High SES	13	0.085	0.048	[−0.009, 0.179]			
Unknown	14	0.172	0.061	[0.052, 0.292]			
**Intervention Design**					6.22	1	0.014
Quasi-experimental	11	0.26	0.085	[0.093, 0.425]			
RCT	55	0.043	0.017	[0.014, 0.080]			
**Subject Area**					4.83	2	0.089
English language arts	31	0.064	0.028	[0.009, 0.118]			
Mathematics	30	0.06	0.026	[0.01, 0.11]			
GPA	5	0.314	0.113	[0.092, 0.536]			
**Report Type**					18.586	1	0.000
Peer-reviewed	64	0.082	0.02	[0.044, 0.121]			
Report	2	−0.07	0.029	[−0.127,−0.012]			

**Table 5:** Subgroup categorical moderator analysis in middle school.

Moderator/Group	k	Point Estimate	Standard Error	95% CI [LL, UL]	Q-Value	df (Q)	*p*-Value
**Students’ SES**					2.423	1	0.12
Low SES	1	0.419	0.193	[0.042, 0.797]			
Unknown	9	0.117	0.023	[0.072, 0.162]			
**Intervention Design**					2.423	1	0.12
Quasi-experimental	1	0.419	0.193	[0.042, 0.797]			
RCT	9	0.117	0.023	[0.072, 0.162]			
**Subject Area**					6.752	3	0.08
English language arts	1	0.146	0.047	[0.054, 0.238]			
Mathematics	3	0.145	0.044	[0.06, 0.231]			
Science	3	0.061	0.028	[0.007, 0.115]			
GPA	3	0.419	0.193	[0.042, 0.797]			
**Report Type**					2.423	1	0.12
Peer-reviewed	1	0.419	0.193	[0.042, 0.797]			
Report	9	0.117	0.023	[0.072, 0.162]			

**Table 6:** Subgroup categorical moderator analysis in mathematic performance.

Moderator/Group	k	Point Estimate	Standard Error	95% CI [LL, UL]	Q-Value	df (Q)	*p*-Value
**Students’ SES**					8.221	2	0.016
Low SES	18	0.061	0.035	[−0.007, 0.129]			
Medium or High SES	6	−0.001	0.049	[−0.096, 0.095]			
Unknown	9	0.139	0.024	[0.092, 0.186]			
**Intervention Design**					0.266	1	0.606
Quasi-experimental	3	0.026	0.095	[−0.16, 0.213]			
RCT	30	0.077	0.025	[0.028, 0.126]			
**Grade Level**					2.847	1	0.092
Elementary	30	0.06	0.026	[0.01, 0.11]			
Middle	3	0.145	0.044	[0.06, 0.231]			
**Report Type**					0.1	1	0.752
Peer-reviewed	29	0.068	0.026	[0.016, 0.119]			
Report	4	0.09	0.065	[−0.038, 0.218]			

**Table 7:** Subgroup categorical moderator analysis in English language arts performance.

Moderator/Group	k	Point Estimate	Standard Error	95% CI [LL, UL]	Q-Value	df (Q)	*p*-Value
**Students’ SES**					4.238	2	0.12
Low SES	40	0.056	0.021	[0.015, 0.098]			
Medium or High SES	13	0.085	0.048	[−0.009, 0.179]			
Unknown	23	0.126	0.026	[0.074, 0.177]			
**Intervention Design**					6.22	1	0.013
Quasi-experimental	12	0.27	0.082	[0.11, 0.43]			
RCT	64	0.063	0.015	[0.033, 0.093]			
**Grade Level**					2.338	1	0.126
Elementary	66	0.075	0.019	[0.038, 0.112]			
Middle	10	0.122	0.024	[0.075 0.168]			
**Report Type**					0.008	1	0.93
Peer-reviewed	65	0.085	0.02	[0.047, 0.124]			
Report	11	0.082	0.031	[0.001, 0.022]			

## Data Availability

No new data were created or analyzed in this study. Data sharing is not applicable to this article.

## Note

1 Ashdown and Bernard (2012), Brackett et al. (2012), Carroll et al. (2020), Challen et al. (2011), Cook et al. (2018), Dougherty and Sharkey (2017), Hanson et al. (2011), Hart et al. (2020), Hennessey and Humphrey (2019), Jones et al. (2011), Kopelman-Rubin et al. (2021), Matsuba et al. (2021), McCormick et al. (2015, 2016, 2021), O’Connor et al. (2014), Panayiotou et al. (2019), Schonfeld et al. (2015), Schonert-Reichl et al. (2015), Tijms et al. (2018), Zhai et al. (2015) indicate studies included in the meta-analysis.
